# Intention to Use Digital Health Among COPD Patients in Europe: A Cluster Analysis

**DOI:** 10.3390/healthcare14020178

**Published:** 2026-01-09

**Authors:** Solomon Getachew Alem, Le Nguyen, Nadia Hipólito, Maelle Spiller, Esther Metting

**Affiliations:** 1 Department of Epidemiology and Biostatistics, Addis Continental Institute of Public Health, Addis Ababa P.O. Box 26751, Ethiopia; 2 Department of Neurology, Erasmus Medical Center, 3015 Rotterdam, The Netherlands; t.t.l.nguyen.1@erasmusmc.nl; 3 Department of Public Health, Erasmus Medical Center, 3015 Rotterdam, The Netherlands; 4 Center for Innovative Care and Health Technology, 2414-016 Leiria, Portugal; nadia.hiplolito@ipleiria.pt; 5 Department of Bioinformatics, University Medical Center Groningen (UMCG), 9700 Groningen, The Netherlands; maelle.spiller@gmx.net; 6 Department of Epidemiology, University Medical Center Groningen (UMCG), 9700 Groningen, The Netherlands; e.i.metting@umcg.nl

**Keywords:** COPD, digital health, self-management, technology acceptance, older adults, digital health interventions

## Abstract

**Background:** Chronic obstructive pulmonary disease (COPD) increasingly strains European health systems amid population ageing. Digital health interventions (DHIs) can reduce hospitalizations and support self-management, yet older patients hesitate to adopt them. Tailored interventions require understanding patient profiles. This study aimed to identify clusters by intention to use DHIs. **Methods:** Between July 2024 and February 2025, 232 COPD patients (mean age 65; 61% female) across seven European countries completed surveys covering sociodemographic and Unified Theory of Technology Acceptance (UTAUT) constructs. Intention to use DHIs was categorized as positive, neutral, or negative. Weighted UTAUT scores were clustered using Gower distance and Partitioning Around Medoids. Associations were visualized with multiple correspondence analysis and heat maps; differences were tested with the chi-square test. **Results:** Intention to adopt DHIs varied across countries, with the highest in the Netherlands. Two clusters emerged. Cluster 1, the ‘balanced hesitant’ group (*n* = 104), showed mixed intentions (38% positive, 40% neutral, 21% negative). Barriers included low performance expectancy and limited digital skills (both *p* < 0.05). Cluster 2, the ‘enthusiastic’ group (*n* = 128), demonstrated strong adoption intentions, with 84% positive intention. Enablers included low effort expectancy and complex disease (*p* < 0.01). Across both clusters, performance expectancy predicted intention. **Conclusions:** Digital health adoption among COPD patients is shaped by psychosocial and digital skill profiles. Hesitant users benefit from expectation-based information about DHIs, digital literacy training and peer support. Enthusiasts require ease of integration. Performance expectancy is a consistent driver of adoption, whereas country-specific factors should guide strategies.

## 1. Introduction

Healthcare systems in Europe are facing growing challenges due to demographic and epidemiological transitions, population aging, a rising prevalence of chronic diseases, and more complex care needs, all of which contribute to higher healthcare costs and workforce pressures [1,2]. Chronic obstructive pulmonary disease (COPD) exemplifies these challenges. As a progressive respiratory disorder associated with substantial morbidity and mortality, COPD remains a major public health priority in Europe [3]. In 2019, the median age-standardized prevalence rate was 3230 per 100,000 among males and 2202 per 100,000 among females, placing COPD among the leading causes of death and disability and imposing a considerable burden on health systems [4]. Furthermore, Exacerbation-related hospitalizations contribute substantially to COPD-related healthcare costs, underscoring the importance of effective and sustainable management strategies beyond conventional facility-based care [5].

Digital health interventions (DHIs), collectively referred to as digital health, offer promising solutions to strengthen COPD management. These include tools such as telemedicine, mobile health applications (mhealth), electronic health records, and remote patient monitoring systems [6]. Evidence demonstrates that digital health interventions can reduce hospitalizations, lower healthcare costs, enhance patient satisfaction, and improve clinical outcomes [7,8,9]. Such benefits are particularly relevant for chronic conditions like COPD, where continuity of care, early detection of exacerbations, and timely clinical support are critical.

Beyond remote monitoring and telemedicine, both clinical and patient-facing decision support systems are integral components of digital health. These systems provide tailored recommendations, alert clinicians to potential risks, and support patients in making informed health decisions, thereby strengthening chronic disease management [10]. In parallel, healthcare delivery is shifting toward more participatory, patient-centered models that emphasize patient engagement and self-management [11]. For older adults living with chronic diseases, digital health tools can support self-management by improving access to health information, facilitating communication with healthcare providers, and enabling remote monitoring and timely interventions [12]. By extending care beyond traditional provider–patient interactions, digital health empowers individuals to better understand their conditions, adhere to treatment plans, and make informed lifestyle choices, with the potential to reduce pressure on healthcare systems [13].

Europe represents a particularly favorable context for the adoption and scaling of DHIs. High internet penetration, widespread mobile connectivity, and robust digital infrastructure, and supportive policy initiatives at the European Union level facilitate the integration of digital technologies into healthcare systems [14,15]. Population-based studies further demonstrate high levels of digital literacy and readiness to use digital health tools among European populations, supporting the feasibility of large-scale DHI implementation in chronic disease management [16,17,18]. However, despite these advantages, significant disparities in healthcare access and outcomes persist across and within European countries, driven by socioeconomic inequalities, geographic variation, and systemic barriers to care [19,20]. Addressing these inequities remains a key public health goal for the region, underscoring the need for digital health strategies that explicitly promote equitable access and outcomes [21]. In parallel, European regulatory frameworks and cross-border digital health initiatives support interoperability and integration across health systems [22]. By contrast, regions with lower connectivity and limited resources often face barriers to implementation, including affordability constraints, infrastructure gaps, and lower acceptance of digital technologies [23,24,25,26]. Taken together, these contextual factors position Europe as a suitable setting for evaluating scalable digital health solutions for COPD management.

Despite these advantages, the adoption of digital health among older adults with chronic respiratory conditions remains limited. Advanced age, lower socioeconomic status, reduced educational attainment, cognitive decline, and limited digital access have all been associated with lower engagement [27,28]. These barriers reflect underlying inequalities in access and engagement with digital technologies, which may exacerbate existing disparities in health outcomes if not addressed proactively [29]. Low digital health literacy further constrains individuals’ ability to benefit from DHIs, raising concerns about equity and inclusivity in digital health programs [30]. Understanding the determinants of digital health acceptance is therefore essential. The Unified Theory of Acceptance and Use of Technology (UTAUT) framework provides a comprehensive approach to examining behavioral intention, emphasizing performance expectancy, effort expectancy, social influence, and facilitating conditions [31,32]. Although not originally developed for healthcare settings, UTAUT has been widely applied in health research and has demonstrated utility in predicting intention to use digital technologies. The framework focuses on the individual user while accounting for demographic characteristics, social influence, and attitude toward technology.

In addition to individual-level factors, recent studies highlight the value of data-driven clustering approaches for capturing heterogeneity in digital health acceptance. By grouping individuals based on shared characteristics such as digital literacy, prior technology experience, and psychosocial factors, clustering enables the identification of meaningful subgroups with distinct adoption patterns [33,34,35]. Such subgrouping helps reveal systematic differences in behaviors, attitudes, and contextual factors, thereby supporting the design of targeted, user-centered interventions. Understanding heterogeneity in technology adoption further supports the development of equitable and sustainable digital health solutions that address the diverse needs of patient populations [36].

Beyond traditional individual-level predictors, utilization-based cluster analysis has been used to identify subgroups of patients with similar health behaviors, technology use patterns, and psychosocial characteristics. In digital health, this method highlights variations in adoption, engagement, and readiness to use digital tools by systematically grouping individuals based on these factors [37,38]. This approach enables the identification of patient subgroups for tailored interventions. Previous studies have applied clustering to characterize multi-morbidity, assess digital literacy, and identify barriers to digital health engagement in older adults [39,40]. Using cluster analysis, one such study revealed distinct patient subgroups with heterogeneous digital health literacy profiles, including variations in digital competencies and access to digital services, highlighting the utility of clustering to inform targeted digital health approaches [40]. These studies underscore its value as an exploratory method to uncover latent patterns that inform context-specific digital health strategies.

This study aims to examine COPD patients’ intention to use digital health interventions using an integrated inferential and cluster-based approach. Specifically, we identify data-driven subgroups based on UTAUT constructs and associated engagement factors and conduct comparative analyses to explore differences in technology acceptance across seven European countries.

## 2. Methods

### 2.1. Study Design, Setting, and Population

This cross-sectional study was conducted across seven European countries. The study is conducted in collaboration with respiratory disease patient organizations in the participating countries. Participant recruitment occurred from July 2024 to February 2025, with patients invited through these organizations to complete a survey, which took approximately 15 min. Eligible participants were adults diagnosed with chronic lung diseases, specifically chronic obstructive pulmonary disease (COPD), who were willing to participate and proficient in reading and writing in one of the following languages: Dutch, German, Spanish, Portuguese, Romanian, or English. The questionnaire was distributed through various patient organizations and healthcare centers. In the Netherlands, Belgium, the UK, and Germany, patients could choose to complete the questionnaire either on paper or online. Participants were asked whether they preferred to receive a link to the online version or a paper copy. Paper questionnaires could be returned in a prepaid envelope at no additional cost. In Spain, Portugal, and Romania, paper distribution was not feasible, so all questionnaires were completed online. The study received ethical approval from the Medical Ethics Committee of the University Medical Center Groningen, Netherlands.

### 2.2. Study Tools

The survey was developed for patients with COPD, typically for a population with lower socioeconomic status. The aim was to make the questionnaire easy to read for COPD patients. The survey consisted of 42 items and contained three parts: (1) demographic questions and questions about digital literacy; (2) items originally developed in a previous study, informed by eight previous focus group discussions conducted in the Netherlands, Belgium, the UK, and Germany, were used to guide the development of the current survey [41], particularly for questions grounded in the UTAUT framework. This section was adapted from a previously validated UTAUT-based instrument, incorporating measures of intention and other core UTAUT constructs; and (3) perceptions and skills: A pragmatic approach was employed to simplify the survey language, enhancing accessibility for participants with limited literacy. This approach, particularly suited for investigating digital health adoption among patients with COPD, was developed in collaboration with a patient panel from the Groningen Research Institute for Asthma and COPD (GRIAC) group at the University Medical Center Groningen.

### 2.3. Outcome Measurement

Digital health acceptance was defined as the intention to use digital health and was assessed with the item “Are you motivated to use digital health?” Responses were recorded on a 5-point Likert scale ranging from 1 (totally disagree) to 5 (totally agree). For analysis, responses are categorized into negative (totally disagree and disagree), neutral, and positive (totally agree and agree).

### 2.4. Predictors

Predictors of digital health acceptance were grouped into four conceptual blocks. The first block, UTAUT constructs, included performance expectancy (2 items), effort expectancy (2 items), and social influence (1 item), all rated on a 3-point Likert scale (1 = disagree to 3 = agree). The second block, perceptions, encompassed attitudes toward the behavior (5 items), self-efficacy (1 item), perceived convenience/timely care (3 items), and perceived data accessibility (2 items), derived from focus group findings and rated on the same 3-point scale. The third block assessed skills, including digital literacy measured using Eurostat’s ICT usage questionnaire (categorized as “Basic” or “Below basic”) and prior experience with at least one digital health tool in the past 12 months (yes/no). The fourth block covered sociodemographic characteristics, living situation, and self-reported physical limitations, most of which were dichotomized for analysis.

### 2.5. Data Analysis for Cluster Development

The dataset was drawn from a multi-country survey conducted between July 2024 and February 2025 among individuals with COPD across seven European countries, initially including 454 patients. The largest group of respondents were from the Netherlands (*n* = 274, 60.4%), followed by Flanders (*n* = 47, 10.4%), the UK (*n* = 41, 9.1%), Romania (*n* = 41, 7.7%), Germany (*n* = 30, 6.6%), and others (*n* = 26, 5.7%). To mitigate this imbalance, a random 25% subsample of Dutch participants was selected, reducing their share to 25.8% [42]. After excluding 12 participants (4.9%) with missing data, the final dataset included 232 participants. Categorical variables were recoded into meaningful levels, numerical variables were standardized, and outliers were retained to preserve data integrity. Analyses were conducted using R version 4.5.1.

### 2.6. Inferential Analysis

Descriptive statistics summarized participant sociodemographic characteristics and key predictors based on the UTAUT framework. Categorical variables were recoded into meaningful levels, and numerical variables were standardized. Cross-country differences and relationships between intention and participant characteristics were examined. Associations between categorical variables and intention to use digital health were assessed using chi-square and Fisher’s exact tests as recommended, with *p*-values < 0.05 considered statistically significant.

### 2.7. Cluster Analysis

**Figure 1:**
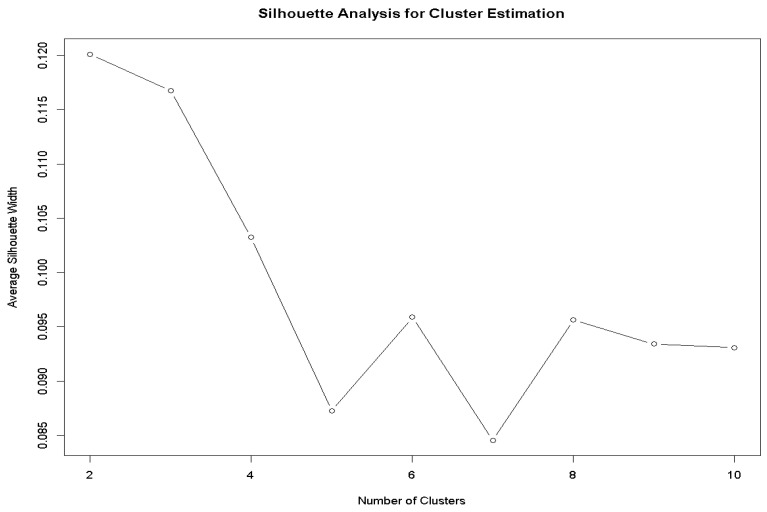
Silhouette analysis for optimal cluster estimation (optimal number of clusters selected as 2).

Cluster analysis was performed using Gower’s dissimilarity metric, which accommodates both categorical and numerical variables. To prioritize digital health-related factors, the digital health variable was assigned a higher weight, resulting in an average Gower distance of 0.53, indicating moderate dissimilarity among observations. Silhouette analysis for k = 2–10 identified two clusters as optimal, with the highest average silhouette width of 0.12 (Figure 1). Based on this, the Partitioning Around Medoids (PAM) algorithm, chosen for its robustness in handling noisy categorical data and interpretability of medoids, identified two clusters comprising 104 and 128 participants. To assess the robustness of the clustering solution, sensitivity analyses were conducted. First, the clustering was repeated, excluding intention to determine whether its inclusion influenced cluster formation, and the cluster solution remained broadly consistent, indicating that structure was not solely driven by intention. Second, the clustering was re-run without variable weighting in the Gower distance, yielding a similar distance structure (average pairwise Gower distance = 0.54), suggesting that the cluster solution was not driven by the weighting scheme. Multiple correspondence analysis (MCA) was also employed to visualize patterns in sociodemographic factors and key UTAUT predictors.

### 2.8. Sample Size Determination

Sample size was determined based on feasibility, prior eHealth adoption studies for exploratory cluster analysis [43]. With 12 clustering variables, a minimum of 120–240 participants were targeted (10–20 cases per variable) to support cluster recovery. The final sample (*n* = 232; two subsamples of *n* = 104 and *n* = 128) exceeded this threshold. Cluster solutions were evaluated for stability using bootstrapping (adjusted Rand index) and internal validity (silhouette width, Calinski–Harabasz index). Post hoc comparisons of eHealth intention between emergent clusters revealed a large difference (Cohen’s h = 0.99, *p* < 0.001), consistent with distinct behavioral profiles.

### 2.9. Multiple Correspondence Analysis

To explore associations between variables and the identified clusters, Multiple Correspondence Analysis (MCA) was conducted [44]. The dataset, comprising only categorical variables recoded from sociodemographic, health, and digital health perception measures, was cleaned, recoded, and formatted into appropriate categories to ensure compatibility with MCA. The cluster variable was treated as a supplementary variable to avoid influencing the construction of the MCA dimensions, ensuring an unbiased representation of the underlying data structure. The analysis retained the first two dimensions, which together accounted for 16.5% of the total variance (Dimension 1: 9.3%; Dimension 2: 7.2%). The stability of the MCA solution was validated using a permutation test (*p* < 0.05).

**Figure 2:**
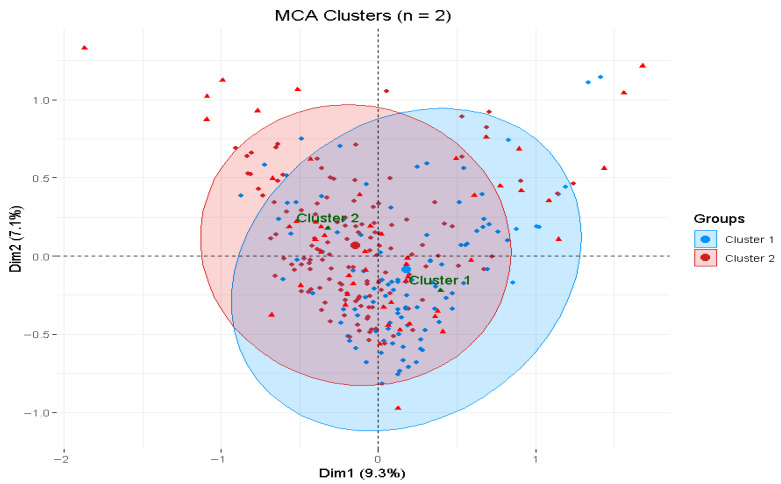
Multiple correspondence analysis biplot for Clusters 1 and 2, showing variance explained by Dimension 1 (9.3%) and Dimension 2 (7.2%). Each point represents an individual observation. Blue circles indicate individuals classified in Cluster 1, while red triangles indicate individuals classified in Cluster 2.

The MCA biplot (Figure 2) illustrates the relationships between variables and the two clusters (Cluster 1 and Cluster 2) derived from the earlier cluster analysis. Cluster 1, depicted by blue points, and Cluster 2, shown by red points, exhibit overlapping distributions, suggesting some shared characteristics. The supplementary cluster variable reveals how demographic and health variables align with these clusters, with the central overlap indicating common traits, while the outer regions reflect distinct differences, such as higher digital health perception scores predominantly associated with Cluster 2.

### 2.10. Outcome Measurements and Analysis

The primary outcome, intention to use digital health, was categorized into negative, neutral, and positive levels and evaluated across the two clusters. Additional covariates, including sociodemographic and UTAUT predictors, were also analyzed. Chi-square tests and Fisher’s exact tests, as recommended, were performed to assess associations and differences between clusters across covariates, providing insights into the distribution and significance of these relationships.

## 3. Results

### 3.1. Sociodemographic Character

#### Cluster Differences in Sociodemographic and UTAUT Predictors

A comparison of participant characteristics between Cluster 1 (*n* = 104) and Cluster 2 (*n* = 128) showed statistically significant differences across several variables (Table 1). A higher proportion of participants in Cluster 2 had a high level of education (60.9%; 78/128) compared to Cluster 1 (21.2%; 22/104), and this difference was statistically significant (*p* < 0.001). Regarding computer use, 57.8% (74/128) of Cluster 2 participants reported no computer use, versus 26.0% (27/104) in Cluster 1 (*p* < 0.001).

Previous experience with digital health was reported by 59.4% (76/128) of Cluster 2, in contrast to 17.3% (18/104) in Cluster 1 (*p* < 0.001). Similarly, 38.3% (49/128) of participants in Cluster 2 reported positive social influence, compared to 22.1% (23/104) in Cluster 1 (*p* < 0.001). Lower effort expectancy was reported by 71.9% (92/128) in Cluster 2 and 15.4% (16/104) in Cluster 1 (*p* < 0.001). For performance expectancy, 39.1% (50/128) of Cluster 2 participants reported negative expectations, compared to 26.9% (28/104) in Cluster 1, and this difference was statistically significant (*p* = 0.041).

Digital literacy was higher in Cluster 2, with 94.5% (121/128) having basic or above literacy, compared to 82.7% (86/104) in Cluster 1 (*p* = 0.004). A greater proportion of participants in Cluster 2 also reported positive voluntariness (65.6%; 84/128) versus Cluster 1 (36.5%; 38/104) (*p* < 0.001). Variables such as sex, work status, country, and health conditions did not show statistically significant differences between clusters.

### 3.2. Cross-Country Differences in Intention to Use Digital Health: Consideration of UTAUT Constructs and Sociodemographic Factors

Cross-country differences were observed in intention to use digital health, with the highest positive intention in the Netherlands (Table 2). The differences across countries were statistically significant (*p* = 0.007).

**Figure 3:**
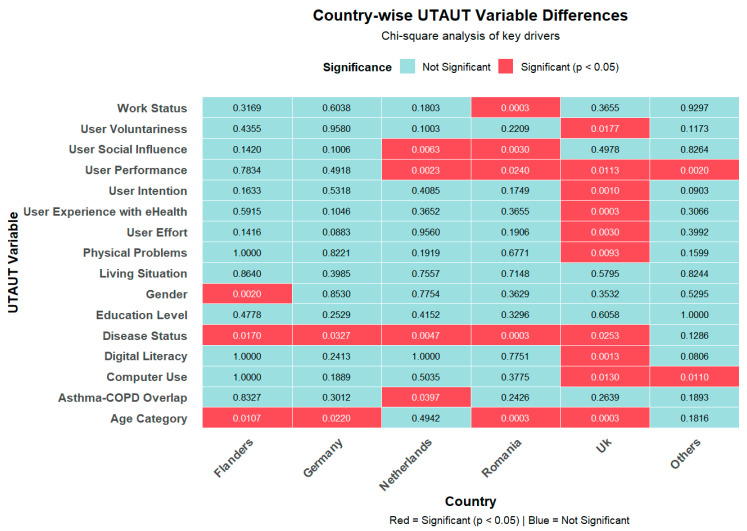
Cross-country differences in intention to use digital health based on UTAUT constructs.

Significant differences in factors associated with intention to use digital health varied across countries. In Flanders, sex was significantly associated with intention to use digital health, with males showing higher acceptance than females (*p* = 0.003). Age was also influential in Flanders (*p* = 0.010), indicating that acceptance differed across age groups (Figure 3).

In Germany, older age groups showed significantly different patterns of acceptance relative to younger individuals (*p* = 0.022). The Netherlands demonstrated several significant associations. Social influence showed a significant relationship with intention to use digital health (*p* = 0.006), indicating that encouragement from others plays a role in technology uptake. Disease status was also significant (*p* = 0.005), as well as performance expectancy (*p* = 0.002), suggesting that perceived usefulness is an important driver of acceptance in this setting.

Romania showed the highest number of significant associations. Age (*p* < 0.001), work status (*p* < 0.001), and disease status (*p* < 0.001) were all strongly linked to intention to use digital health. Social influence (*p* = 0.003) and performance expectancy (*p* = 0.024) were also significant, further reinforcing the importance of perceived usefulness and interpersonal motivation.

In the United Kingdom, participants with experience using digital technologies were more likely to have higher acceptance of digital health (*p* < 0.001). Physical problems (*p* = 0.009), age (*p* < 0.001), disease status (*p* = 0.025), digital literacy (*p* = 0.001), voluntariness (*p* = 0.017), and performance expectancy (*p* = 0.011) were also significantly associated, indicating a broader set of determinants compared to other countries.

Among participants from countries categorized as “Others,” which includes Spain, Portugal, and Belgium, computer use (*p* = 0.011) and performance expectancy (*p* = 0.002) showed significant links with intention to use digital health, demonstrating that capability and perceived usefulness remain core drivers in settings outside the primary study countries. Overall, performance expectancy and disease status emerged as the most consistently significant factors across countries, while age category and social influence showed important, but more location-specific effects.

### 3.3. User Intention to Use Digital Health Across Clusters

Cluster 2 had a significantly higher proportion of positive intentions (107, 83.59%) compared to Cluster 1 (40, 38.46%), while Cluster 1 had more neutral (42, 40.38%) and negative (22, 21.15%) responses. The Pearson chi-square test confirmed this strong association (*p* < 0.001), indicating distinct sentiment patterns between clusters.

### 3.4. Cluster Profiles of Digital Health Intention and UTAUT Predictors

Associations between intention to use digital health (Negative: *n* = 36, 15.52%; Neutral: *n* = 49, 21.12%; Positive: *n* = 147, 63.36%) and UTAUT predictors were examined within each cluster using chi-square tests (*p* < 0.05). Cluster 1 (*n* = 104) showed a balanced intention distribution: 22 participants (21.15%) reported Negative, 42 (40.38%) Neutral, and 40 (38.46%) Positive intentions. Cluster 2 (*n* = 128) was dominated by Positive intention (*n* = 107, 83.59%), with smaller Negative (*n* = 14, 10.94%) and Neutral (*n* = 7, 5.47%) subgroups (Supplementary Table S1).

#### 3.4.1. Balanced-Hesitant Subgroup

In Cluster 1, intention to use digital health was significantly associated with perceived social influence (*p* < 0.001), performance expectancy (*p* < 0.001), and digital literacy (*p* = 0.016). Participants with Negative intentions were most likely to report low social influence (40.91% Negative, 45.45% Neutral, and 13.64% Positive) and lower performance expectancy (72.73% Neutral, 27.27% Positive). In contrast, those with Positive intentions were more likely to perceive stronger social influence (40% Positive) and demonstrate better digital literacy (92.50% with basic or above literacy). Neutral-intention participants tended to fall between these groups, suggesting that moderate perceptions of social influence and digital skills may correspond to uncertain digital health engagement (Supplementary Table S1).

#### 3.4.2. Positive Intention Subgroup

In Cluster 2, intention to use digital health showed significant associations with disease type (*p* = 0.005), prior digital health experience (*p* = 0.004), perceived social influence (*p* < 0.001), effort expectancy (*p* < 0.001), and performance expectancy (*p* < 0.001). Participants with Positive intentions were most often those with asthma–COPD overlap (22.43%) or COPD (57.00%), prior digital health experience (63.16% Positive), and higher perceptions of social influence (43.93% Positive). They also demonstrated stronger beliefs in ease of use (78.57% Positive effort expectancy) and usefulness (53.27% Neutral, 0.93% Positive performance expectancy). Conversely, Negative intentions were more common among individuals with COPD only (57.14%), limited digital health experience (29.41% Negative experience), and low effort expectancy (64.00% Negative). Demographic variables, including sex and age category, did not significantly influence intention in either cluster, emphasizing the dominance of behavioral and perceptual factors (Supplementary Table S1).

## 4. Discussion

This multi-country study examined intention to use digital health interventions among patients with COPD across seven European countries using an integrated inferential and cluster-based analytical approach grounded in the UTAUT framework. Two distinct patient groups emerged, reflecting contrasting psychosocial and behavioral patterns related to digital health adoption. Cluster 1 demonstrated a balanced but hesitant stance toward adoption, whereas Cluster 2 showed strong enthusiasm and readiness to engage. Importantly, demographic factors such as age and country of residence did not significantly predict cluster membership, indicating that acceptance among older adults with chronic conditions is shaped by factors beyond traditional population characteristics. This finding is consistent with prior research highlighting the limited explanatory power of demographics alone [45] and underscores the importance of developing context-specific strategies to enhance digital health uptake rather than relying on demographic-based assumptions.

Cluster 1 demonstrated mixed intentions toward digital health, with hesitation linked to limited digital literacy, lower education, and weak social influence. Prior studies show that when social influence is strong, particularly from family or healthcare providers, facilitates digital health uptake among older adults [46,47,48,49] and enhances mhealth engagement [50,51]. In this study, weak social influence contributed to reluctance in Cluster 1, suggesting that strengthening peer and professional support mechanisms may help offset adoption barriers. Interventions targeting modifiable factors such as digital literacy training and structured peer support have been shown to improve uptake and engagement and may be particularly effective for this group. This profile is illustrated in Supplementary Figure S1 as the “hesitant user”.

In contrast, Cluster 2 reflected a digitally confident and resilient group, consistent with evidence that higher education, prior technology use, and employment facilitate digital health adoption [52]. Participants who were employed or living with others also showed greater readiness to adopt digital tools, suggesting that social connectedness and continued occupational engagement promote comfort with technology. Conversely, the small subgroup with negative intentions tended to include retirees, individuals with lower education, and those from contexts where technology use is less embedded in daily life, potentially reflecting perceived irrelevance or lower confidence in digital solutions. Key facilitators of digital health adoption in this cluster included prior experience with digital health, higher performance and effort expectancy, strong social influence, and the presence of more complex disease conditions such as asthma-COPD overlap. These findings align with previous studies showing that greater illness burden can motivate individuals to adopt technologies that support disease self-management [53,54,55]. Similarly, familiarity with technology and perceptions of ease of use reinforce sustained adoption, consistent with evidence that experience and usability are critical for long-term engagement among older adults [56,57]. This profile is illustrated in Supplementary Figure S1 as the “enthusiastic adopter.”

Differences in digital health intention across countries appear linked to variations in UTAUT constructs, particularly performance expectancy, effort expectancy, social influence, and digital literacy, rather than to demographic characteristics [58,59]. As shown in Figure 3, significant contrasts were observed mainly for user performance, intention, effort, and social influence, suggesting that national contexts shape these psychosocial determinants more than demographic factors. This pattern aligns with prior evidence indicating that cross-country disparities in technology adoption are driven less by infrastructure and more by contextual differences in perceived usefulness, ease of use, and social endorsement [60].

### 4.1. Implications of the Study

While the UTAUT framework is widely used in digital health research, this study extends its application to a multi-country European COPD population. Our findings confirm the relevance of core UTAUT predictors, including performance expectancy, effort expectancy, social influence, and voluntariness. In addition, cluster-based analyses show that psychosocial and behavioral factors, rather than demographic characteristics, primarily differentiate adoption profiles, with digital literacy, education, and computer use emerging as relevant contextual determinants of digital health adoption.

Taken together, these findings indicate that digital health adoption among patients with COPD and related respiratory conditions depends more on psychosocial and skill-related factors than on demographics alone. For clinical practice, this highlights the importance of understanding patients’ individual needs for information, support, and confidence-building when implementing digital health interventions. Assessing digital literacy, prior experience, and perceived usefulness may help clinicians tailor recommendations and improve patient engagement.

From a digital health design perspective, the identification of distinct user profiles has important implications for tailoring interventions. For individuals in Cluster 1, our findings indicate that expectations and social influence are the most salient modifiable predictors of digital health acceptance. While certain determinants, such as prior experience and educational level, are less amenable to change, several key factors can be addressed through targeted design and implementation strategies. In particular, effort expectancy may be enhanced by providing adequate guidance and ongoing practical support, while performance expectancy can be strengthened by clearly communicating what the technology can and cannot do. In addition, leveraging social influence, for example, by actively involving partners, family members, or other significant others in the adoption process, appears to be a promising approach. Finally, maintaining voluntariness of use, such as by ensuring the availability of non-digital or parallel care options, may help reduce resistance and support sustained engagement with digital health technologies. In contrast, individuals in Cluster 2, who demonstrate higher motivation, experience, and digital readiness, may benefit more from advanced and personalized digital health features that build on their existing capabilities and positive expectations.

Additionally, these findings highlight the potential of digital health interventions to reduce disparities by improving access to information, supporting self-management, and ensuring continuity of care [61]. Addressing these equity-related factors is critical to ensure that digital health adoption benefits all patient groups, particularly those facing barriers related to digital literacy, socioeconomic status, or limited access to technology. At the policy level, these results suggest that efforts to scale digital health for chronic disease management should emphasize capacity building, user-centered design, and supportive implementation environments rather than focusing solely on access or infrastructure.

### 4.2. Future Directions

Future research should build on these findings by developing and evaluating interventions that target modifiable psychosocial and skill-related factors to help patients overcome barriers to digital health use, improve participation, and maximize impact. Recognizing different patient groups can inform tailored, targeted strategies, such as digital literacy support for hesitant users and usability-focused programs for motivated users. Longitudinal studies are also needed to assess whether intention translates into sustained engagement, and country-specific or context-sensitive analyses could clarify how cultural, organizational, and health system factors shape adoption, supporting locally adapted and scalable implementation strategies.

### 4.3. Limitations of the Study

This study has several limitations. First, its cross-sectional design precludes causal inference between predictors and intention. Second, although the survey was available in multiple languages, cultural nuances in technology perception across countries may not have been fully captured, which could have influenced the observed cross-country variation in adoption patterns. Third, the use of single- or two-item measures for constructs such as self-efficacy, social influence, and performance expectancy may limit sensitivity at the subscale level, though the overall intention-to-use scale remains valid for assessing general technology acceptance. Finally, although the silhouette width indicated weak separation, the two-cluster solution captured meaningful behavioral distinctions. Despite modest cohesion, the clusters provide useful insights for exploratory interpretation and can inform the design of targeted digital health interventions. Therefore, these cluster patterns should be considered exploratory and interpreted with caution when guiding intervention strategies.

## 5. Conclusions

Digital health adoption in COPD and related chronic respiratory conditions depends primarily on psychosocial factors and digital skills rather than demographics alone. Key modifiable drivers such as digital literacy, effort expectancy, and performance expectancy, and social support offer clear targets for interventions to boost confidence and engagement among hesitant patients. While adoption patterns were largely consistent across countries, cross-national differences highlight the influence of local culture and healthcare systems, necessitating culturally tailored strategies. Cluster-based approaches can thus enhance equity, participation, and program impact for older adults throughout European settings. Although demographic characteristics are less amenable to change, several key factors can be addressed through targeted design and implementation strategies. In particular, effort expectancy may be enhanced by providing adequate guidance, tailored information, and ongoing practical support, while performance expectancy can be strengthened by clearly communicating what the technology can and cannot do. Future studies should incorporate more diverse groups and larger samples to strengthen generalizability and capture broader variation in adoption patterns.

## Figures and Tables

**Table 1:** Comparison of sociodemographic and UTAUT predictors by cluster membership (*n* = 232).

Variable	Categories	Cluster 1 (*n* = 104)	Cluster 2 (*n* = 128)	Total	*p*-Value
Sex	Male	43 (41.4%)	47 (36.7%)	90 (38.79%)	0.472
	Female	61 (58.7%)	81 (63.3%)	142 (61.21%)	
Age Group	29–55	12 (11.5%)	28 (21.9%)	40 (17.24%)	0.082
	55–64	22 (21.2%)	34 (26.6%)	56 (24.14%)	
	65–79	60 (57.7%)	57 (44.5%)	117 (50.43%)	
	80+	10 (9.6%)	9 (7.0%)	19 (8.19%)	
Work Status	Employed	23 (22.1%)	32 (25.0%)	55 (23.71%)	0.854
	Unemployed	1 (1.0%)	3 (2.3%)	4 (1.72%)	
	Medically unfit	16 (15.4%)	18 (14.1%)	34 (14.66%)	
	Retired	59 (56.7%)	71 (55.5%)	130 (56.03%)	
	Other	5 (4.8%)	4 (3.1%)	9 (3.88%)	
Education	Low	68 (65.4%)	34 (26.6%)	102 (43.97%)	<0.001 ***
	Middle	14 (13.5%)	16 (12.5%)	30 (12.93%)	
	High	22 (21.2%)	78 (60.9%)	100 (43.10%)	
Computer Use at Work	None	27 (26.0%)	74 (57.8%)	101 (43.53%)	<0.001 ***
	Occasional	33 (31.7%)	37 (28.9%)	70 (30.17%)	
	Frequent	44 (42.3%)	17 (13.3%)	61 (26.29%)	
Living Situation	Lives alone	27 (26.0%)	47 (36.7%)	74 (31.90%)	0.08
	Doesn’t live alone	77 (74.0%)	81 (63.3%)	158 (68.10%)	
Country	Netherlands	26 (25.0%)	34 (26.6%)	25 (10.78%)	0.315
	Flanders	21 (20.2%)	25 (19.5%)	46 (19.83%)	
	Germany	16 (15.4%)	12 (9.4%)	28 (12.07%)	
	Romania	14 (13.5%)	21 (16.4%)	60 (25.86%)	
	UK	20 (19.2%)	18 (14.1%)	35 (15.09%)	
	Other	7 (6.7%)	18 (14.1%)	38 (16.38%)	
Health Conditions	COPD	68 (65.4%)	74 (57.8%)	142 (61.21%)	0.748
	Asthma	9 (8.7%)	11 (8.6%)	20 (8.62%)	
	Asthma/COPD overlap	16 (15.4%)	28 (21.9%)	44 (18.97%)	
	Cystic Fibrosis	1 (1.0%)	1 (0.8%)	2 (0.86%)	
	Other	10 (9.6%)	14 (10.9%)	24 (10.34%)	
Physical Problems	Yes	69 (66.4%)	97 (75.8%)	166 (71.55%)	0.113
	No	35 (33.7%)	31 (24.2%)	66 (28.45%)	
Digital health Experience	Negative	8 (7.7%)	10 (7.8%)	18 (7.76%)	<0.001 ***
	Neutral	78 (75.0%)	42 (32.8%)	120 (51.72%)	
	Positive	18 (17.3%)	76 (59.4%)	94 (40.51%)	
Social Influence	Negative	16 (15.4%)	44 (34.4%)	120 (51.72%)	<0.001 ***
	Neutral	65 (62.5%)	35 (27.3%)	94 (40.52%)	
	Positive	23 (22.1%)	49 (38.3%)	60 (25.86%)	
Effort Expectancy	Negative	30 (28.9%)	17 (13.3%)	100 (43.10%)	<0.001 ***
	Neutral	58 (55.8%)	19 (14.8%)	72 (31.03%)	
	Positive	16 (15.4%)	92 (71.9%)	36 (15.52%)	
Performance Expectancy	Negative	28 (26.9%)	50 (39.1%)	49 (21.12%)	0.041 *
	Neutral	68 (65.4%)	75 (58.6%)	147 (63.36%)	
	Positive	8 (7.7%)	3 (2.3%)	47 (20.26%)	
Digital Literacy	Basic or above	86 (82.7%)	121 (94.5%)	207 (89.22%)	0.004 **
	No basic literacy	18 (17.3%)	7 (5.5%)	25 (10.77%)	
Voluntariness	Negative	56 (53.9%)	29 (22.7%)	85 (36.63%)	<0.001 ***
	Neutral	10 (9.6%)	15 (11.7%)	25 (10.77%)	
	Positive	38 (36.5%)	84 (65.6%)	122 (52.58%)	

*p*-values are denoted as follows: *** *p* < 0.001, ** *p* < 0.01, * *p* < 0.05.

**Table 2:** Cross-country differences in intention to use digital health (*p* = 0.007).

Countries	Negative (%)	Neutral (%)	Positive (%)	Total (%)
Netherlands	10 (27.78)	16 (32.65)	34 (23.13)	60 (25.86)
Flanders	3 (8.33)	10 (20.41)	33 (22.45)	46 (19.83)
Romania	3 (8.33)	5 (10.20)	27 (18.37)	35 (15.09)
Germany	3 (8.33)	8 (16.33)	17 (11.56)	28 (12.07)
UK	13 (36.11)	9 (18.37)	16 (10.88)	38 (16.38)
Others	4 (11.11)	1 (2.04)	20 (13.61)	25 (10.78)

## Data Availability

The data presented in this study are available upon request from the corresponding author.

## Supplementary Materials

The following supporting information can be downloaded at: https://www.mdpi.com/article/10.3390/healthcare14020178/s1, Figure S1. Profile of User Clusters Based on Digital Health Intention and Characteristics. Table S1. Intention Across Clusters with Different UTAUT Predictors.

## Abbreviations

The following abbreviations are used in this manuscript:

COPD	Chronic Obstructive Pulmonary Disease
GRIAC	Groningen Research Institute for Asthma and COPD
DHI	Digital Health Intervention
UTAUT	Unified Theory of Acceptance and Use of Technology
PAM	Partitioning Around Medoids
MCA	Multiple Correspondence Analysis
METc	Medical Ethics Committee
UMCG	University Medical Center Groningen
ARI	Adjusted Rand Index
CH Index	Calinski–Harabasz Index

## References

[1] Demographic Change in Europe—October 2023—Eurobarometer Survey. https://europa.eu/eurobarometer/surveys/detail/3112.

[2] Gianfredi V., Nucci D., Pennisi F., Maggi S., Veronese N., Soysal P. (2025). Aging, longevity, and healthy aging: The public health approach. Aging Clin. Exp. Res..

[3] Agarwal A.K., Raja A., Brown B.D. (2025). Chronic Obstructive Pulmonary Disease. StatPearls.

[4] Marshall D.C., Al Omari O., Goodall R., Shalhoub J., Adcock I.M., Chung K.F., Salciccioli J.D. (2022). Trends in prevalence, mortality, and disability-adjusted life-years relating to chronic obstructive pulmonary disease in Europe: An observational study of the global burden of disease database, 2001–2019. BMC Pulm. Med..

[5] Bollmeier S.G., Hartmann A.P. (2020). Management of chronic obstructive pulmonary disease: A review focusing on exacerbations. Am. J. Health Syst. Pharm..

[6] Hazra S., Bora K.S. (2025). Capitalization of digital healthcare: The cornerstone of emerging medical practices. Intell. Pharm..

[7] Ezeamii V.C., Okobi O.E., Wambai-Sani H., Perera G.S., Zaynieva S., Okonkwo C.C., Ohaiba M.M., William-Enemali P.C., Obodo O.R., Obiefuna N.G. (2024). Revolutionizing Healthcare: How Telemedicine Is Improving Patient Outcomes and Expanding Access to Care. Cureus.

[8] Mishra V., Stuckler D., McNamara C.L. (2024). Digital Interventions to reduce hospitalization and hospital readmission for chronic obstructive pulmonary disease (COPD) patient: Systematic review. BMC Digit. Health.

[9] Achelrod D., Schreyögg J., Stargardt T. (2017). Health-economic evaluation of home telemonitoring for COPD in Germany: Evidence from a large population-based cohort. Eur. J. Health Econ..

[10] Sutton R.T., Pincock D., Baumgart D.C., Sadowski D.C., Fedorak R.N., Kroeker K.I. (2020). An overview of clinical decision support systems: Benefits, risks, and strategies for success. npj Digit. Med..

[11] Hickmann E., Richter P., Schlieter H. (2022). All Together Now—Patient Engagement, Patient Empowerment, and Associated Terms in Personal Healthcare. BMC Health Serv. Res..

[12] Erku D., Khatri R., Endalamaw A., Wolka E., Nigatu F., Zewdie A., Assefa Y. (2023). Digital Health Interventions to Improve Access to and Quality of Primary Health Care Services: A Scoping Review. Int. J. Env. Res. Public Health.

[13] Park Y., Kim E.-J., Park S., Lee M. (2025). Digital Health Intervention Effect on Older Adults with Chronic Diseases Living Alone: Systematic Review and Meta-Analysis of Randomized Controlled Trials. J. Med. Internet Res..

[14] TheGlobalEconomy.com [Internet] Mobile Network Coverage in Europe. https://www.theglobaleconomy.com/rankings/Mobile_network_coverage/Europe/.

[15] Digital Health and Care—Public Health—European Commission. https://health.ec.europa.eu/ehealth-digital-health-and-care/digital-health-and-care_en.

[16] Admassu W., Gorems K. (2024). Analyzing health service employees’ intention to use e-health systems in southwest Ethiopia: Using UTAUT-2 model. BMC Health Serv. Res..

[17] Bente B.E., Van Dongen A., Verdaasdonk R., van Gemert-Pijnen L. (2024). eHealth implementation in Europe: A scoping review on legal, ethical, financial, and technological aspects. Front. Digit. Health.

[18] de Veer A.J.E., Peeters J.M., Brabers A.E., Schellevis F.G., Rademakers J.J.J., Francke A.L. (2015). Determinants of the intention to use e-Health by community dwelling older people. BMC Health Serv. Res..

[19] Coppini V., Ferraris G., Ferrari M.V., Dahò M., Kirac I., Renko I., Monzani D., Grasso R., Pravettoni G. (2024). Patients’ perspectives on cancer care disparities in Central and Eastern European countries: Experiencing taboos, misinformation and barriers in the healthcare system. Front. Oncol..

[20] Health Equity Status Report Initiative. https://www.who.int/europe/initiatives/health-equity-status-report-initiative.

[21] Bucciardini R., Zetterquist P., Rotko T., Putatti V., Mattioli B., De Castro P., Napolitani F., Giammarioli A.M., Kumar B.N., Nordström C. (2023). Addressing health inequalities in Europe: Key messages from the Joint Action Health Equity Europe (JAHEE). Arch. Public Health.

[22] The European Health Data Space (EHDS). https://www.european-health-data-space.com/.

[23] Mamuye A., Nigatu A.M., Chanyalew M.A., Amor L.B., Loukil S., Moyo C., Quarshie S., Antypas K., Tilahun B. (2023). Facilitators and Barriers to the Sustainability of eHealth Solutions in Low- and Middle-Income Countries: Descriptive Exploratory Study. JMIR Form. Res..

[24] Archer N., Lokker C., Ghasemaghaei M., DiLiberto D. (2021). eHealth Implementation Issues in Low-Resource Countries: Model, Survey, and Analysis of User Experience. J. Med. Internet Res..

[25] Mahmoud K., Jaramillo C., Barteit S. (2022). Telemedicine in Low- and Middle-Income Countries During the COVID-19 Pandemic: A Scoping Review. Front. Public Health.

[26] Poulsen A., Hickie I.B., Alam M., Wilson C.E., LaMonica H.M. (2025). Access to Mobile Health in Lower Middle-Income Countries: A Review. Health Technol..

[27] Bertolazzi A., Quaglia V., Bongelli R. (2024). Barriers and facilitators to health technology adoption by older adults with chronic diseases: An integrative systematic review. BMC Public Health.

[28] Metting E., van Luenen S., Baron A.-J., Tran A., van Duinhoven S., Chavannes N.H., Hevink M., Lüers J., Kocks J. (2023). Overcoming the Digital Divide for Older Patients with Respiratory Disease: Focus Group Study. JMIR Form. Res..

[29] König L.M., Krukowski R.A., Kuntsche E., Busse H., Gumbert L., Gemesi K., Neter E., Mohamed N.F., Ross K.M., John-Akinola Y.O. (2023). Reducing intervention- and research-induced inequalities to tackle the digital divide in health promotion. Int. J. Equity Health.

[30] Fitzpatrick P.J. (2023). Improving health literacy using the power of digital communications to achieve better health outcomes for patients and practitioners. Front. Digit. Health.

[31] Xue L., Rashid A.M., Ouyang S. (2024). The Unified Theory of Acceptance and Use of Technology (UTAUT) in Higher Education: A Systematic Review. Sage Open.

[32] Dwivedi Y.K., Rana N.P., Jeyaraj A., Clement M., Williams M.D. (2019). Re-examining the Unified Theory of Acceptance and Use of Technology (UTAUT): Towards a Revised Theoretical Model. Inf. Syst. Front..

[33] Peng C., Chen Z., Zhou H., Dai C., Yuan H., Gao Y., Wang F., Liang Z. (2025). Quantitative CT and COPD: Cluster analysis reveals five distinct subtypes with varying exacerbation risks. BMC Pulm. Med..

[34] Wang C., Yu F., Cao Z., Huang K., Chen Q., Geldsetzer P., Zhao J., Zheng Z., Bärnighausen T., Yang T. (2025). Exploring COPD Patient Clusters and Associations with Health-Related Quality of Life Using a Machine Learning Approach: A Nationwide Cross-Sectional Study. Engineering.

[35] Chikhanie Y.A., Bailly S., Amroussa I., Veale D., Hérengt F., Verges S. (2022). Clustering of COPD patients and their response to pulmonary rehabilitation. Respir. Med..

[36] van Deursen A.J., van Dijk J.A. (2019). The first-level digital divide shifts from inequalities in physical access to inequalities in material access. New Media Soc..

[37] Nittas V., Zecca C., Kamm C.P., Kuhle J., Chan A., von Wyl V. (2023). Digital health for chronic disease management: An exploratory method to investigating technology adoption potential. PLoS ONE.

[38] Tartaglia J., Jaghab B., Ismail M., Hänsel K., Meter A.V., Kirschenbaum M., Sobolev M., Kane J.M., Tang S.X. (2024). Assessing Health Technology Literacy and Attitudes of Patients in an Urban Outpatient Psychiatry Clinic: Cross-Sectional Survey Study. JMIR Ment. Health.

[39] Violán C., Roso-Llorach A., Foguet-Boreu Q., Guisado-Clavero M., Pons-Vigués M., Pujol-Ribera E., Valderas J.M. (2018). Multimorbidity patterns with K-means nonhierarchical cluster analysis. BMC Fam. Pract..

[40] Andersen M.H., Hermansen Å., Dahl K.G., Lønning K., Meyer K.B., Vidnes T.K., Wahl A.K. (2024). Profiles of health literacy and digital health literacy in clusters of hospitalised patients: A single-centre, cross-sectional study. BMJ Open.

[41] Metting E.I. (2024). Cultural differences in technology acceptance of COPD patients oh cool: A crosscountry focusgroup study. Eur. Respir. J..

[42] Alamolhoda M., Ayatollahi S.M.T., Bagheri Z. (2017). A comparative study of the impacts of unbalanced sample sizes on the four synthesized methods of meta-analytic structural equation modeling. BMC Res. Notes.

[43] Black A.D., Car J., Pagliari C., Anandan C., Cresswell K., Bokun T., McKinstry B., Procter R., Majeed A., Sheikh A. (2011). The Impact of eHealth on the Quality and Safety of Health Care: A Systematic Overview. PLoS Med..

[44] Florensa D., Mateo-Fornés J., Solsona F., Pedrol Aige T., Mesas Julió M., Piñol R., Godoy P. (2022). Use of Multiple Correspondence Analysis and K-means to Explore Associations Between Risk Factors and Likelihood of Colorectal Cancer: Cross-sectional Study. J. Med. Internet Res..

[45] Lin X.Y., Moxley J., Sharit J., Czaja S.J. (2025). Beyond the Digital Divide: Factors Associated with Adoption of Technologies Related to Aging in Place. J. Appl. Gerontol..

[46] Pan C.-C., De Santis K.K., Muellmann S., Hoffmann S., Spallek J., Barnils N.P., Ahrens W., Zeeb H., Schüz B. (2025). Sociodemographics and Digital Health Literacy in Using Wearables for Health Promotion and Disease Prevention: Cross-Sectional Nationwide Survey in Germany. J. Prev..

[47] Shao Y., Yang X., Chen Q., Guo H., Duan X., Xu X., Yue J., Zhang Z., Zhao S., Zhang S. (2025). Determinants of digital health literacy among older adult patients with chronic diseases: A qualitative study. Front. Public Health.

[48] Shams-Ghahfarokhi Z. (2025). Challenges in health and technological literacy of older adults: A qualitative study in Isfahan. BMC Geriatr..

[49] Tan M.M.T., Wong R.S.H., Goh W.J.W.H., Hilal S. (2025). Facilitators and Barriers for Use of Digital Technology in Chronic Disease Management. Sci. Rep..

[50] Kanahara N., Hirabayashi M., Mamada T., Nishimoto M., Iyo M. (2018). Combination therapy of electroconvulsive therapy and aripiprazole for dopamine supersensitivity psychosis. Schizophr. Res..

[51] Hepburn J., Williams L., McCann L. (2025). Barriers to and Facilitators of Digital Health Technology Adoption Among Older Adults with Chronic Diseases: Updated Systematic Review. JMIR Aging.

[52] Tuitert I., Marinus J.D., Dalenberg J.R., van ’t Veer J.T. (2024). Digital Health Technology Use Across Socioeconomic Groups Prior to and During the COVID-19 Pandemic: Panel Study. JMIR Public Health Surveill..

[53] Zheng J., Zhao J., Li B., Sun J., Zeng X. (2025). Determinants of Chronic Disease Patients’ Intention to Use Internet Diagnosis and Treatment Services: Based on the UTAUT2 Model. Front. Digit. Health.

[54] Turja T., Jylhä V., Rosenlund M., Kuusisto H. (2025). Beyond early and late adopters: Reimagining health technology readiness through health-related circumstances. Sustain. Futures.

[55] Mahajan Y., Agarwal P., Chintamani A.A., Pahurkar R., Bhinde H.N., Sharma V. (2025). Perceived Ease of Use and Health Literacy as Determinants of mHealth App Usage Among Older Adults in India: A SEM Approach. Discov. Soc. Sci. Health.

[56] Yu W., Ji Y., Li Z., Wang K., Jiang X., Chang C. (2025). Study on the “digital divide” in the continuous utilization of Internet medical services for older adults: Combination with PLS-SEM and fsQCA analysis approach. Int. J. Equity Health.

[57] Jokisch M.R., Schmidt L.I., Doh M. (2022). Acceptance of digital health services among older adults: Findings on perceived usefulness, self-efficacy, privacy concerns, ICT knowledge, and support seeking. Front. Public Health.

[58] Schmitz A., Díaz-Martín A.M., Yagüe Guillén M.J. (2022). Modifying UTAUT2 for a cross-country comparison of telemedicine adoption. Comput. Hum. Behav..

[59] Levin-Zamir D., Van den Broucke S., Bíró É., Bøggild H., Bruton L., De Gani S.M., Finbråten H.S., Gibney S., Griebler R., Griese L. (2025). Measuring Digital Health Literacy and Its Associations with Determinants and Health Outcomes in 13 Countries. Front. Public Health.

[60] Venkatesh V., Sykes T.A., Zhang X. (2020). ICT for Development in Rural India: A Longitudinal Study of Women’s Health Outcomes. MIS Q..

[61] Qoseem I.O., Okesanya O.J., Olaleke N.O., Ukoaka B.M., Amisu B.O., Ogaya J.B., Lucero-Prisno D.E. (2024). Digital health and health equity: How digital health can address healthcare disparities and improve access to quality care in Africa. Health Promot. Perspect..

