# Supplementary material for: Intention to Use Digital Health Among COPD Patients in Europe: A Cluster Analysis
Source: Healthcare (Basel). 2026 Jan 9;14(2):178. doi: 10.3390/healthcare14020178 (PMC12841130; doi:10.3390/healthcare14020178)
Supplement: Supplementary file 1 [file healthcare-14-00178-s001.zip › Supplementary Table S1.pdf]

Supplimenarty Table S1. Intention Across Clusters with Different UTAUT Predictors.

		Cluster 1					cluster 2				
Variable	Category	Negative	Neutral	Positive	Total	(p-value)	Negative	Neutral	Positive	Total	(p-value)
Sex	Man	8 (36.36%)	19 (45.24%)	16 (40.00%)	43	(0.772)	7 (50.00%)	0 (0.00%)	40 (37.38%)	47	(0.076)
	Woman	14 (63.64%)	23 (54.76%)	24 (60.00%)	61		7 (50.00%)	7 (100.00%)	67 (62.62%)	81	
Age category	18-55	0 (0.00%)	5 (11.90%)	7 (17.50%)	12	(0.139)	1 (7.14%)	2 (28.57%)	25 (23.36%)	28	(0.670)
	55-64	2 (9.09%)	10 (23.81%)	10 (25.00%)	22		4 (28.57%)	1 (14.29%)	29 (27.10%)	34	
	65-79	18 (81.82%)	24 (57.14%)	18 (45.00%)	60		7 (50.00%)	4 (57.14%)	46 (42.99%)	57	
	80-100	2 (9.09%)	3 (7.14%)	5 (12.50%)	10		2 (14.29%)	0 (0.00%)	7 (6.54%)	9	
Work Status	I have a job	5 (22.73%)	7 (16.67%)	11 (27.50%)	23	(0.739)	3 (21.43%)	2 (28.57%)	27 (25.23%)	32	(0.165)
	Looking for a job	0 (0.00%)	1 (2.38%)	0 (0.00%)	1		1 (7.14%)	1 (14.29%)	1 (0.93%)	3	

	Medically unfit to work	3 (13.64%)	8 (19.05%)	5 (12.50%)	16		0 (0.00%)	0 (0.00%)	18 (16.82%)	18	
	Retired	14 (63.64%)	24 (57.14%)	21 (52.50%)	59		10 (71.43%)	4 (57.14%)	57 (53.27%)	71	
	Doing something else	0 (0.00%)	2 (4.76%)	3 (7.50%)	5		0 (0.00%)	0 (0.00%)	4 (3.74%)	4	
Education	Low education	14 (63.64%)	22 (52.38%)	32 (80.00%)	68	(0.100)	2 (14.29%)	1 (14.29%)	31 (28.97%)	34	(0.405)
	Middle education	4 (18.18%)	8 (19.05%)	2 (5.00%)	14		3 (21.43%)	0 (0.00%)	13 (12.15%)	16	
	High education	4 (18.18%)	12 (28.57%)	6 (15.00%)	22		9 (64.29%)	6 (85.71%)	63 (58.88%)	78	
Computer use	No / Not applicable	3 (13.64%)	15 (35.71%)	9 (22.50%)	27	(0.304)	6 (42.86%)	4 (57.14%)	64 (59.81%)	74	(0.795)
	Occasionally use computer	7 (31.82%)	11 (26.19%)	15 (37.50%)	33		5 (35.71%)	2 (28.57%)	30 (28.04%)	37	
	Use computer most of the day	12 (54.55%)	16 (38.10%)	16 (40.00%)	44		3 (21.43%)	1 (14.29%)	13 (12.15%)	17	
Living Situation	Live alone	9 (40.91%)	8 (19.05%)	10 (25.00%)	27	(0.164)	4 (28.57%)	5 (71.43%)	38 (35.51%)	47	(0.129)
	Do not live alone	13 (59.09%)	34 (80.95%)	30 (75.00%)	77		10 (71.43%)	2 (28.57%)	69 (64.49%)	81	

	Others	1 (4.55%)	1 (2.38%)	5 (12.50%)	7		3 (21.43%)	0 (0.00%)	15 (14.02%)	18	
	Flanders	3 (13.64%)	10 (23.81%)	8 (20.00%)	21		0 (0.00%)	0 (0.00%)	25 (23.36%)	25	
Country of Origin	Germany	3 (13.64%)	7 (16.67%)	6 (15.00%)	16	(0.166)	0 (0.00%)	1 (14.29%)	11 (10.28%)	12	(0.098)
	Netherlands	6 (27.27%)	12 (28.57%)	8 (20.00%)	26		4 (28.57%)	4 (57.14%)	26 (24.30%)	34	
	Romania	1 (4.55%)	4 (9.52%)	9 (22.50%)	14		2 (14.29%)	1 (14.29%)	18 (16.82%)	21	
	UK	8 (36.36%)	8 (19.05%)	4 (10.00%)	20		5 (35.71%)	1 (14.29%)	12 (11.21%)	18	
Asthma COPD overlap	No	19 (86.36%)	34 (80.95%)	35 (87.50%)	88	(0.691)	10 (71.43%)	7 (100.00%)	83 (77.57%)	100	(0.309)
	Asthma-COPD overlap	3 (13.64%)	8 (19.05%)	5 (12.50%)	16		4 (28.57%)	0 (0.00%)	24 (22.43%)	28	
disease	COPD	16 (72.73%)	26 (61.90%)	26 (65.00%)	68	(0.891)	8 (57.14%)	5 (71.43%)	61 (57.01%)	74	
	Asthma	1 (4.55%)	3 (7.14%)	5 (12.50%)	9		0 (0.00%)	0 (0.00%)	11 (10.28%)	11	(0.005)*

	Asthma/COPD overlap syndrome	3 (13.64%)	8 (19.05%)	5 (12.50%)	16		4 (28.57%)	0 (0.00%)	24 (22.43%)	28	
	Cystic Fibrosis	0 (0.00%)	1 (2.38%)	0 (0.00%)	1		0 (0.00%)	1 (14.29%)	0 (0.00%)	1	
	Other	2 (9.09%)	4 (9.52%)	4 (10.00%)	10		2 (14.29%)	1 (14.29%)	11 (10.28%)	14	
Physical problems	No	9 (40.91%)	16 (38.10%)	10 (25.00%)	35	(0.328)	3 (21.43%)	1 (14.29%)	27 (25.23%)	31	(0.780)
	Yes	13 (59.09%)	26 (61.90%)	30 (75.00%)	69		11 (78.57%)	6 (85.71%)	80 (74.77%)	97	
User Experience in digital health	Negative	4 (18.18%)	3 (7.14%)	1 (2.50%)	8	(0.221)	4 (28.57%)	0 (0.00%)	6 (5.61%)	10	(0.004)*
	Neutral	16 (72.73%)	31 (73.81%)	31 (77.50%)	78		7 (50.00%)	1 (14.29%)	34 (31.78%)	42	
	Positive	2 (9.09%)	8 (19.05%)	8 (20.00%)	18		3 (21.43%)	6 (85.71%)	67 (62.62%)	76	
User Social influence	Negative	9 (40.91%)	5 (11.90%)	2 (5.00%)	16	(0.000)*	12 (85.71%)	4 (57.14%)	28 (26.17%)	44	(0.000)*
	Neutral	10 (45.45%)	33 (78.57%)	22 (55.00%)	65		1 (7.14%)	2 (28.57%)	32 (29.91%)	35	

	Positive	3 (13.64%)	4 (9.52%)	16 (40.00%)	23		1 (7.14%)	1 (14.29%)	47 (43.93%)	49	
User effort	Negative	10 (45.45%)	13 (30.95%)	7 (17.50%)	30	(0.078)	9 (64.29%)	1 (14.29%)	7 (6.54%)	17	(0.000)*
	Neutral	10 (45.45%)	25 (59.52%)	23 (57.50%)	58		2 (14.29%)	1 (14.29%)	16 (14.95%)	19	
	Positive	2 (9.09%)	4 (9.52%)	10 (25.00%)	16		3 (21.43%)	5 (71.43%)	84 (78.50%)	92	
User Performance	Negative	0 (0.00%)	7 (16.67%)	21 (52.50%)	28	(0.000)*	0 (0.00%)	1 (14.29%)	49 (45.79%)	50	(0.000)*
	Neutral	16 (72.73%)	34 (80.95%)	18 (45.00%)	68		12 (85.71%)	6 (85.71%)	57 (53.27%)	75	
	Positive	6 (27.27%)	1 (2.38%)	1 (2.50%)	8		2 (14.29%)	0 (0.00%)	1 (0.93%)	3	
Digital literacy	No basic digital literacy	8 (36.36%)	7 (16.67%)	3 (7.50%)	18	(0.016)*	3 (21.43%)	0 (0.00%)	4 (3.74%)	7	(0.019)*
	Basic or above digital literacy	14 (63.64%)	35 (83.33%)	37 (92.50%)	86		11 (78.57%)	7 (100.00%)	103 (96.26%)	121	
User Voluntariness	Negative	12 (54.55%)	24 (57.14%)	20 (50.00%)	56	(0.134)	1 (7.14%)	2 (28.57%)	26 (24.30%)	29	(0.677)
	Neutral	2 (9.09%)	7 (16.67%)	1 (2.50%)	10		2 (14.29%)	1 (14.29%)	12 (11.21%)	15	

	Positive	8 (36.36%)	11 (26.19%)	19 (47.50%)	38		11 (78.57%)	4 (57.14%)	69 (64.49%)	84	
