# Supplementary figures and images for: Intention to Use Digital Health Among COPD Patients in Europe: A Cluster Analysis
Source: Healthcare (Basel). 2026 Jan 9;14(2):178. doi: 10.3390/healthcare14020178 (PMC12841130; doi:10.3390/healthcare14020178)

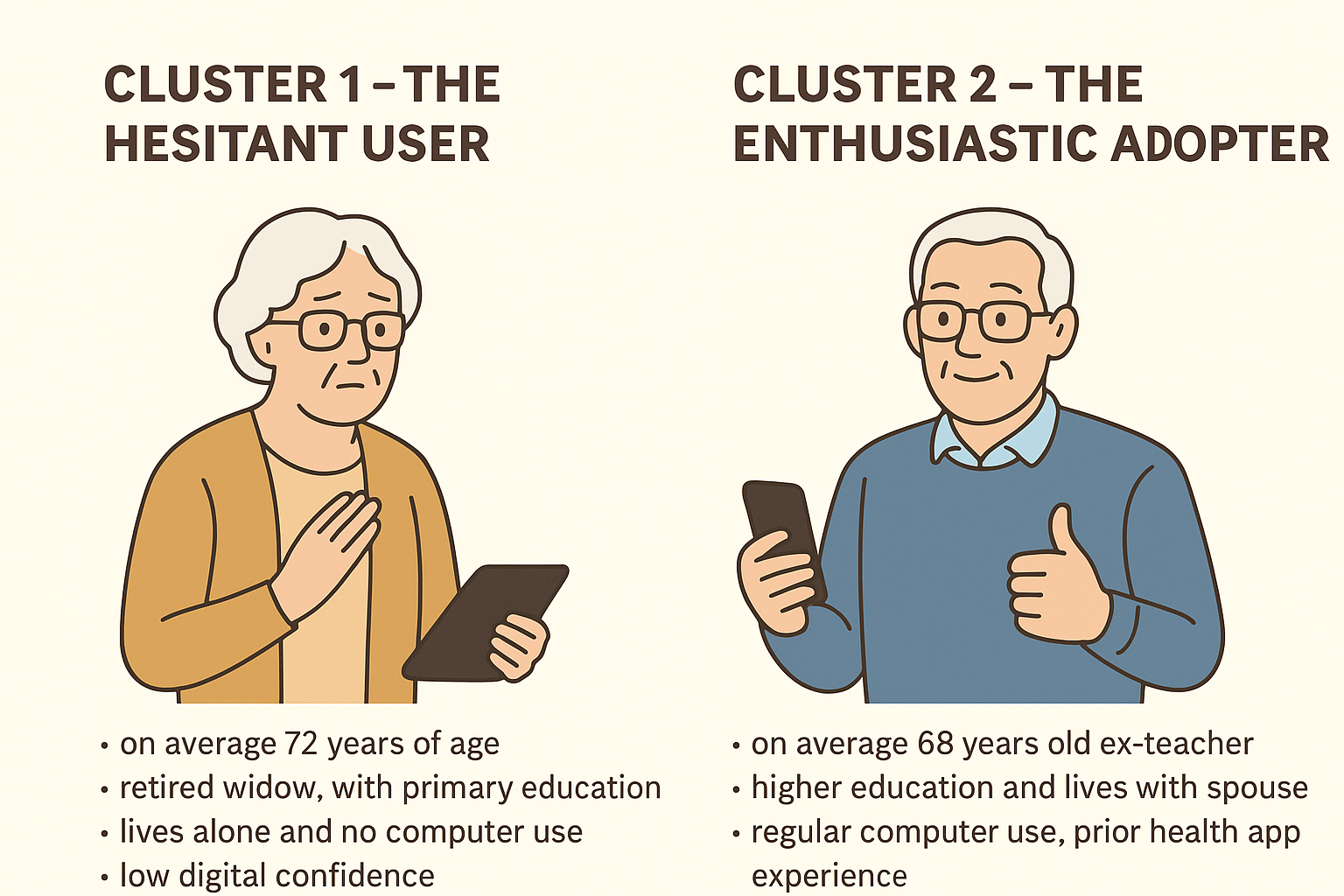

Supplement: Supplementary file 1 [file healthcare-14-00178-s001.zip › Supplementary Figure S1 Profile of User Clusters Based on Digital Health Intention and Characteristics.png]
